# Fast and Slow-Growing Management Systems: Characterisation of Broiler Caecal Microbiota Development throughout the Growing Period

**DOI:** 10.3390/ani10081401

**Published:** 2020-08-12

**Authors:** Laura Montoro-Dasi, Arantxa Villagra, María de Toro, María Teresa Pérez-Gracia, Santiago Vega, Clara Marin

**Affiliations:** 1 Instituto de Ciencia y Tecnología Animal, Universidad Politécnica de Valencia, 46022 Valencia, Spain; laura.montoro@outlook.com; 2 Centro de Calidad Avícola y Alimentación Animal de la Comunidad Valenciana (CECAV), 12539 Castellón, Spain; 3 Centro de Investigación y Tecnología Animal, Instituto Valenciano de Investigaciones Agrarias, 12400 Castellón, Spain; villagra_ara@gva.es; 4 Plataforma de Genómica y Bioinformática, Centro de Investigación Biomédica de La Rioja, 26006 La Rioja, Spain; mthernando@riojasalud.es; 5 Área de Microbiología, Departamento de Farmacia, Instituto de Ciencias Biomédicas, Facultad de Ciencias de la Salud, Universidad Cardenal Herrera-CEU, CEU Universities, Avenida Seminario s/n, 46113 Moncada, Spain; teresa@uchceu.es; 6 Departamento de Producción y Sanidad Animal, Salud Pública Veterinaria y Ciencia y Tecnología de los Alimentos, Instituto de Ciencias Biomédicas, Facultad de Veterinaria, Universidad Cardenal Herrera-CEU, CEU Universities, Avenida Seminario s/n, 46113 Moncada, Spain; svega@uchceu.es

**Keywords:** broiler, growing period, microbiota, biomarker, 16S rRNA analysis

## Abstract

**Simple Summary:**

This study was conducted to characterise the caecal microbiota in two broiler management systems (fast and slow-growing) during the growing period, using 16S rRNA sequencing analysis. Because of the essential role of the caecal bacteria in poultry health and productivity, these data could be considered as a biomarker of health status and will make it possible to evaluate different treatments applied in animals. The main results demonstrated that microbiota is in constant development throughout the growing period for both management systems, and the most abundant bacteria groups are related to better productive performance and intestinal health.

**Abstract:**

Caecal microbiota and its modulation play an important role in poultry health, productivity and disease control. Moreover, due to the emergence of antimicrobial-resistant bacteria, society is pressing for a reduction in antibiotic administration by finding effective alternatives at farm level, such as less intensified production systems. Hence, the aim of this study was to characterise the caecal microbiota in two different broiler management systems, fast and slow-growing, using 16S rRNA sequencing analysis. To this end 576 broilers were reared in two different management systems (fast and slow-growing). Results showed that *Firmicutes* represented the dominant phylum for both systems. At the onset, *Proteobacteria* was the second prevalent phylum for fast and slow-growing breeds, outnumbering the *Bacteroidetes*. However, during the rest of the production cycle, *Bacteroidetes* was more abundant than *Proteobacteria* in both groups. Finally, regardless of the management system, the most predominant genera identified were *Oscillospira* spp., *Ruminococcus* spp., *Coprococcus* spp., *Lactobacillus* spp. and *Bacteroides* spp. In conclusion, fast and slow-growing broiler microbiota are in constant development throughout rearing, being relatively stable at 21 days of age. Regarding the genus, it should be noted that the three most abundant groups for both systems, *Ruminococcus* spp., *Lactobacillus* spp. and *Bacteroides* spp., are related to better productive performance and intestinal health.

## 1. Introduction

Microbiota is defined as the microbial community, including commensal, symbiotic and pathogenic microorganisms, which colonise different areas of animals and have an important influence on animal health, productivity and disease control [1,2,3,4,5,6,7,8,9]. Hence, the presence of beneficial microbiota plays an important role in production, protection from pathogens, control of epithelial cell proliferation and differentiation, detoxification (controlling the behavioural and neurological functions of the host) and modulation of the immune system [6,9,10].

Principal factors affecting the microbiota are age, breed, maternal elements, sex, diet, housing, hygiene, temperature, litter, antibiotic administration and gastrointestinal location [6,11]. Referring to the last factor mentioned, the caecum is described as the organ with the greatest taxonomic diversity and abundance, which retains food for the longest period, with the greatest water absorption, and is responsible for urea regulation and carbohydrates fermentation [6].

Moreover, due to the emergence of antimicrobial-resistant bacteria, society is pressing for a reduction in antibiotic administration by finding effective alternatives to control infectious diseases at farm level [12,13,14,15]. Some of these alternatives are feed additives (prebiotics, probiotics, symbiotics, organic acids, enzymes, phytogenics and metals), alternative medical treatments (antibacterial vaccines, immunomodulatory agents, antimicrobial peptides and bacteriophages) and, finally, different, less intensified broiler management systems [16,17,18,19,20,21,22]. Although the beneficial effects of many of these alternatives have been demonstrated in vitro, the general consensus is that the effect of these products depends on the farm, farmer management and animal characteristics, such as the breed selected [11,14,23].

The variability obtained in different studies highlights the need to know, under production conditions, how the microbiota evolves, which could assist in decision-making in situ, especially at critical moments of the production period. For example, when the broiler reaches the farm from the incubator, an adaptation moment that will mark the development of the production cycle [9,11,24,25,26]; at the stage when the immune and digestive system is already mature, and therefore, will determine the potential of the breed in terms of growth and conversion [8,27,28]; or at the end of the cycle, a key moment, as it is the step before the animals are transferred to the slaughterhouse. The microbiota has been seen to be an important source of external and internal contamination of the carcass by bacteria of such great importance as *Salmonella* and *Campylobacter* during loading, transport and slaughter [29,30,31,32]. Therefore, having a broad knowledge of this composition throughout the cycle can help the sector choose the different control measures to be applied during rearing, which enhance the presence of beneficial microorganisms, as well as the immune system, and can control and even eliminate the presence of pathogenic microorganisms at critical moments in the production cycle [6,9,32]. However, today there is still a need to develop cost-effective and straightforward molecular techniques that can be used for this purpose at field level.

In this context, the aim of this study was to characterise the caecal microbiota in two different broiler management systems, fast and slow-growing, during their respective growing periods, using 16S rRNA sequencing analysis.

## 2. Materials and Methods

In this experiment, all animals were handled according to the principles of animal care published by Spanish Royal Decree 53/2013 [33]. All protocols were approved by the Ethical Review Panel of the Directorate-General for Agriculture, Fisheries and Livestock from the Valencian Community by the code 2018/VSC/PEA/0067.

### 2.1. Experiment Design

The study was performed in an experimental poultry house in the Centre for Animal Research and Technology (CITA, in its Spanish acronym (Valencian Institute for Agrarian Research, IVIA, Segorbe, Spain)). To this end, 576 broilers (males and females) provided from the same hatchery were randomly housed in two identical poultry rooms (replicates A and B) and 288 animals were housed in each room (144 fast and 144 slow-growing breeds). In addition, animals were distributed in 24 pens (12 pens for each group, fast and slow-growing broiler management system) in a final stocking density of 35 kg/m^2^ for fast-growing management system, and in a final stocking density of 17 kg/m^2^ for slow-growing management system, with wood shavings as bedding material. Two management systems were evaluated: fast and slow-growing, and two commercial breeds were used (Ross^®^ and Hubbard^®^, respectively). The fast-growing management is characterised by using an efficient feed conversion and good meat yield breed [34], with the appropriate feed, and an early slaughter age (42 days). In contrast, the slow-growing management system is a less intensified type of production, focused on the criteria of animal welfare and absence of antibiotics [35], with a different feed and a later slaughter age (63 days).

To simulate the real conditions of broiler production, the houses were supplied with programmable electric lighting, automated electric heating and forced ventilation. The environmental temperature was set at 32 °C on arrival day and gradually reduced to 19 °C by 41 days post hatch.

The birds received drinking water and were fed ad libitum. Nutritional and product safety analysis were assessed before the arrival of animals in the Poultry Quality and Animal Feed Centre of the Valencia Region (CECAV, in its Spanish acronym (Centro de Calidad Avícola y Alimentación Animal de la Comunidad Valenciana, Castellón, Spain)). Two different age commercial diets were offered to the animals: from arrival until 21 days post hatch, chicks were fed a pelleted starter diet, and from 21 days old to the slaughter day a pelleted grower diet was offered to animals. The diets for each management system were formulated to meet each breed’s metabolic requirements and to provide equal nutrient profiles [36]. The starter diet was the same for both breeds (Camperbroiler iniciación, Alimentación Animal Nanta, Spain), while the grower feed was the standard diet specific for each one: A-32 broiler and A-80 Pollos crecimiento (Alimentación Animal Nanta, Spain) for the fast and slow-growing breeds, respectively. Nutritional composition of the diets has been detailed in Table 1. Only one batch of feed per age (starter and grower) was manufactured. Moreover, no coccidiostats or antimicrobials were added to either diet, and high biosecurity levels were maintained in the experimental poultry house during the rearing. Mortality rates and diarrhoea presence were recorded daily. Finally, animals were weighed at weekly intervals and feed consumption per pen was recorded.

### 2.2. Sample Collection

To assess the development of microbiota composition throughout the growing period, animals from each experimental group were randomly selected and caecal samples were collected at different times of the growing period: on arrival day (1 day), at mid-period (21 days for both groups) and before slaughter (42 days of age in fast-growing, and 63 days in slow-growing). On arrival day, 30 animals per group (fast or slow-growing) were selected and sampled just before being assigned to the houses (*n* = 30/group). Later, at mid-period, and before slaughter, caecal samples from 30 animals per group and house were collected again at each sampling moment (*n* = 60/group/sampling moment). Caecal samples were taken individually and placed in sterile jars. The samples were processed immediately after collection.

### 2.3. DNA Extraction

Caecal content was removed and homogenised. On the first day of rearing, five pools of six animals from each experimental group were prepared (*n* = 5/group). Then, for the mid and end period, five pools of six animals from each group and house were made (*n* = 10/group/sampling moment). DNA of the pool content was extracted according to the manufacturer’s instructions (QIAamp Power Fecal DNA kit, Qiagen, Hilden, Germany) and frozen at −80 °C for shipment to the Centre for Biomedical Research of La Rioja (CIBIR, in its Spanish acronym, Logroño, Spain).

### 2.4. 16S rRNA Sequencing Analysis

First, all samples received were analysed in a Fragment Analyzer (Genomic DNA 50 Kb kit, AATI) to ensure their integrity. Additionally, the initial DNA concentration was measured by means of a Qubit fluorometer (dsDNA HS Assay kit, Invitrogen). From 12.5 ng of DNA (evaluated in Qubit) of each sample, the library was prepared following the instructions of the 16S rRNA Metagenomic Sequencing Library Preparation (Illumina) protocol [37]. Primer sequences cover the V3–V4 regions of the 16S rRNA gene. The following primers also include the Illumina adapters: 16S Amplicon PCR Forward Primer = 5′ (TCGTCGGCAGCGTCAGATGTGTATAAGAGACAGCCTACGGGNGGCWGCAG) and 16S Amplicon PCR Reverse Primer = 5′ (GTCTCGTGGGCTCGGAGATGTGTATAAGAGACAGGACTACHVGGGTATCTAATCC). The sequencing run was performed in a MiSeq (Illumina) system in 2 × 300 bp format.

The quality of the raw unprocessed reads was evaluated using the FastQC software [38]. After the removal of adapters by Trim Galore [39], the quality of clean reads was re-evaluated with FastQC. Then, because the fragments sequenced for each of the samples were overlapped in their central region, the V3–V4 region of the 16S rRNA gene was partially reconstructed into fragments of approximately 550–580 bp. The OTU (Operational Taxonomic Unit) picking and analysis was performed with QIIME (v1.9.1) pipeline [40], following the methodology “pick open reference OTUs” against the taxonomy reference base Greengenes 13.8 at 97% nucleotide identity. Finally, InteractiVenn software was used for Venn diagram construction [41].

Calculation of the alpha diversity indexes was done by QIIME (v1.9.1), which generates multiple rarefactions on the OTU table at different sequencing depths, calculates the alpha diversity indexes at each depth, and finally coheres the data, generating rarefaction graphs for each index. To Identify OTUs with differential abundance in this study, the analysis was performed using two tests: a non-parametric analysis (Kruskal–Wallis) and a parametric test (MetagenomeSeq). In both cases, analysis set out from the standardised and filtered table of OTUs to eliminate those OTUs that may be spurious. Analysis was carried out at three taxonomic levels: Phylum, Genera and OTU. Then, the alpha diversity indexes were statistically compared between groups of samples through the Python script “compare_alpha_diversity.py” included in the QIIME v1.9.1 package. It performs a two-sample t-test by using by default, as in our case, a non-parametric (Monte Carlo) method and permutation value of 999. The t-test value and a *p*-value (Bonferroni correction) were obtained for each couple of defined groups. In this study, a rarefaction depth of 72,060 reads was selected for this analysis [42].

Beta diversity was evaluated based on indices or coefficients of similarity, dissimilarity or distance between the samples from qualitative (presence/absence of OTUs) or quantitative (proportional abundance of each OTU) data. The OTU filtering and normalisation from the OTU table was performed using the QIIME v1.9.1 protocol. A threshold of 0.01% was applied, meaning that the OTU sequences with an abundance below the 0.01% are assigned as spurious sequences, and therefore removed from the analysis. The OTU table normalisation, applying the Cumulative Sum Scaling (CSS) method through the MetagenomeSeq package was chosen as an alternative to the rarefaction one, according to previous studies [43,44]. In QIIME’s metagenomics protocol, beta diversity was measured through a distance or dissimilarity matrix between each pair of samples. This matrix was visualised with Principal Coordinate Analysis (PCoA) graphs in 2D and 3D, which allow analysis of the distance between each pair of samples.

Moreover, to analyse the statistical significance of sample groupings by using beta diversity distance matrices, the “compare_categories.py” Qiime v1.9.1 script was used. This script, which uses the R vegan and ape packages, allows analysis of the strength and statistical significance of sample groupings. Several methods are available, and two of them were selected for this study: ANOSIM and Adonis. Both methods were applied to the three different calculated matrices (Bray–Curtis, Unweighted Unifrac and Weighted Unifrac).

### 2.5. Data Availability

Bioproject: PRJNA612272.

BioSample: SAMN14365530: Fast and slow-growing broiler breeds. Caecal microbiota characterisation.

## 3. Results

During this study, a total of 50 caecal pools (25 per experimental group) were collected, processed and sequenced. No clinical signs were observed during rearing, and the productive parameters obtained were in accordance with the breed standards (Table 2). There were no statistical differences between replicates (*p*-value > 0.05).

### 3.1. 16 rRNA Profiling of Fast and Slow-Growing Management Systems

The MiSeq sequencing of the 50 samples produced a total of 14,143,246 sequencing reads with an average of 282,864.9 reads per sample. Quality and chimera filtering produced a total of 12,661,675 filtered reads with an average of 253,233.5 reads per sample and ranging from 109,447 to 356,331 reads.

**Figure 1:**
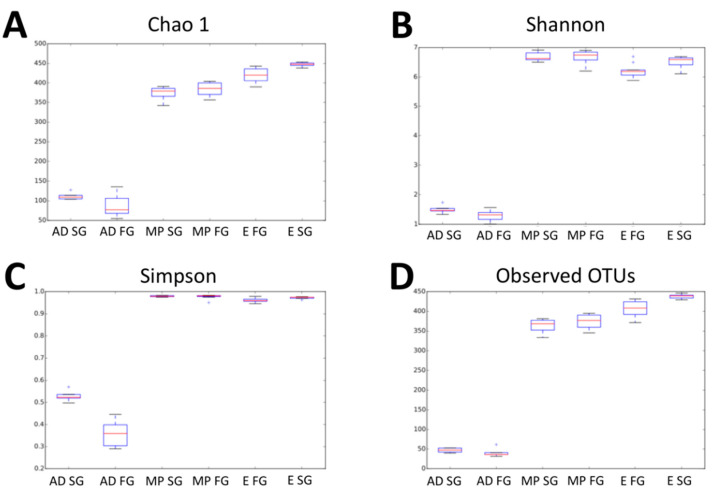
Evaluation of alpha diversity in fast and slow-growing management systems using different calculation measures. (**A**): Chao 1. (**B**): Shannon. (**C**): Simpson. (**D**): Observed OUTs. AD SG: slow-growing breed at arrival day; AD FG: fast-growing breed at arrival day; MP SG: slow-growing breed at mid-period; MP FG: fast-growing breed at mid-period; E FG: fast-growing breed at the end of the growing period; E SG: slow-growing breed at the end of the growing period.

Assessment of rarefaction curves based on the Chao1, Shannon, Simpson and Observed OTUs biodiversity indexes calculated for the six sequence read groups (day-old chicks, mid-period and slaughter day results for fast and slow-growing management systems) indicated that four of the curves tended to reach a plateau (Tables S1–S4). However, samples from groups 1 and 2 (day-old chicks from both groups) are at the limit of the rarefaction, leaving a rarefaction number of 72,060 reads (Figure 1).

The Chao1 alpha diversity index reveals a notable difference between the caecal microbiota depending on the moment of sampling (arrival, mid, end period) (Table 3). For the fast-growing management system, statistically significant differences (*p*-value < 0.05) were found between sampling moments. Samples from day-old chicks (88.3) displayed a lower level of complexity of the microbiota compared to that found at mid-period (384.4), and samples from mid-period animals displayed a lower level of complexity than the samples from the end of the growing period (420.3). Similarly, for the slow-growing management system, alpha diversity index increased throughout the growing period with statistically significant differences between sampling moments, with a result of 111.9, 373.8 and 447.2 on arrival day, at mid-period and at the end of the growing period, respectively.

### 3.2. Differential Gut Microbiota Composition

Inspection of predicted taxonomic profiles at phylum level for all samples based on a MetagenomeSeq parametric test is summarised in Table 4. This analysis showed that *Firmicutes* represented the dominant phylum of the caecal community in both management systems at all sampling times in the production cycle (*p*-value < 0.05). At the onset of the growing period, *Proteobacteria* was the second prevalent phylum for fast and slow-growing breeds, outnumbering the *Bacteroidetes* phylum. However, during the rest of the production cycle, the *Bacteroidetes* phylum was more abundant than *Proteobacteria* in both groups.

For the fast-growing management system, there were statistically significant differences between the phyla prevalence and the time of sampling (arrival day, mid-period and end period). *Proteobacteria* and *Bacteroidetes* phyla were more abundant at the arrival day (36.4% and 5%, respectively). However, *Firmicutes* was the most prevalent phylum at mid-period (95.1%).

For the slow-growing management system, *Bacteroidetes* (5.7% at arrival day) and *Firmicutes* (95.2% at mid-period) showed the same pattern as in the fast-growing breed. However, statistically significant differences were shown between day-old chicks and the mid-period percentage of *Proteobacteria* (32.8% and 1.2%, respectively), which subsequently remained stable until the end of the cycle (1.7%).

**Figure 2:**
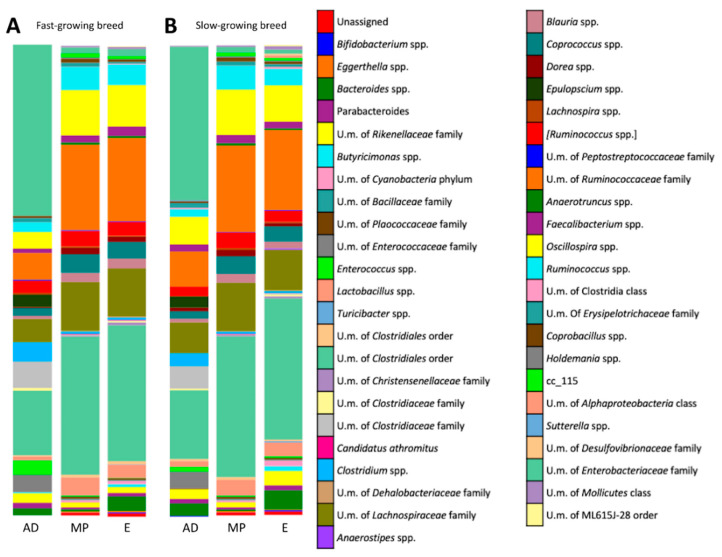
Taxonomic analysis at genus level throughout the growing period. (**A**): Evolution of genera throughout the growing period for fast-growing management system (AD: arrival day, MP: mid-period, E: end). (**B**): Evolution of genera throughout the growing period for slow-growing management system (AD: arrival day, MP: mid-period, E: end).

Furthermore, in this study 46 taxa were identified at genus level (Figure 2). Regardless of the management system and time point, the most predominant genera identified were *Oscillospira* spp. (7.5%), *Ruminococcus* spp. (3.6%), *Coprococcus* spp. (2.9%), *Lactobacillus* spp. (2.5%) and *Bacteroides* spp. (2.0%). In order to further identify microbiota composition for both breeds, we focused on 33 genera, which were shown to be present at an average relative abundance of more than 0.5% in at least one sample group [45].

In addition, it is important to highlight that 75% (24/32), 93% (40/43) and 97.8% (45/46) are common genera for both experimental groups at the beginning, mid- and end period, respectively (the different genera are detailed in Table S5).

For the fast-growing management system, the results of the genera analysis are shown in Table 5. At arrival day, predominant bacteria of microbiota were Unclassified members (U. m.) of the *Enterobacteriaceae* family (36.4%), U. m. of the *Clostridiaceae* family (6.2%), U. m. of the *Ruminococcaceae* family (5.7%), U. m. of the *Lachnospiraceae* family (4.9%), *Clostridium* spp. (4.1%), U. m. of the *Enterococcaceae* family (3.7%), *Oscillospira* spp. (3.5%) and *Enterococcus* spp. (3.0%). At mid-period, the predominant genera in caecal samples were U. m. of the *Ruminococcaceae* family (18.1%), U. m. of the *Lachnospiraceae* family (10.4%), *Oscillospira* spp. (9.6%), *Coprococcus* spp. (4.0%), *Lactobacillus* spp. (3.9%) and *[Ruminococcus]* spp. (3.3%). Finally, at the end of the growing period, the most prevalent bacteria were U. m. of the *Ruminococcaceae* family (17.7%), U. m. of *Lachnospiraceae* family (10.2%), *Oscillospira* spp. (8.8%), *Coprococcus* spp. (3.5%) and *Bacteroides* spp. (3.1%).

For the slow-growing management system, the results of the genera analysis are shown in Table 6. The pattern for day-old chicks was similar to that observed at this sampling time for the fast-gowing group. The most abundant bacteria were U. m. of the *Enterobacteriaceae* family (32.6%), U. m. of the *Ruminococcaceae* family (7.5%), U. m. of the *Lachnospiraceae* family (6.5%), *Oscillospira* spp. (5.8%), U. m. of the *Clostridiaceae* family (4.8%) and U. m. of the *Enterococcaceae* family (3.6%). At mid-period, predominant genera were U. m. of the *Ruminococcaceae* family (18.4%), U. m. of the *Lachnospiraceae* family (10.3%), *Oscillospira* spp. (9.6%), *Coprococcus* spp. (3.8%), *Lactobacillus* spp. (3.4%) and *Ruminococcus* spp. (3.3%). Lastly, at slaughter day, U. m. of the *Ruminococcaceae* family (17.0%) were the most abundant bacteria, followed by U. m. of the *Lachnospiraceae* family (8.6%), *Oscillospira* spp. (7.7%), *Coprococcus* spp. (3.2%), *Bacteroides* spp. (4.1%) and *Parabacteroides* spp. (3.1%).

**Figure 3:**
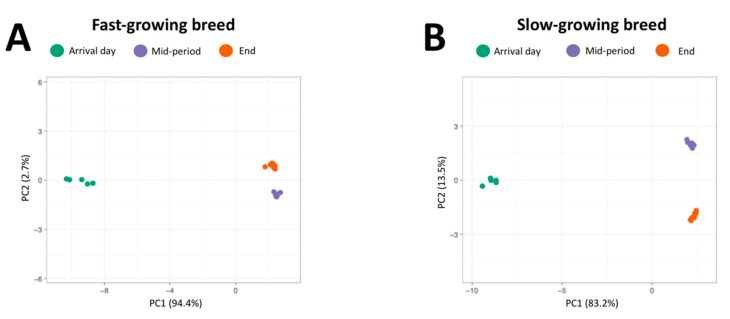
Evaluation of the beta diversity based on Bray–Curtis dissimilarity between sampling moments (arrival day, mid-period and end of the rearing period) for each management system. (**A**): Beta diversity represented by a Principal Coordinate Analysis (PCoA) graphic for fast-growing management system. (**B**): Beta diversity represented by PCoA graphic for slow-growing management system.

Finally, to assess differences in microbiota between sampling moments, beta diversity was analysed based on Bray–Curtis dissimilarity, Weighted UniFrac and Unweighted UniFrac indexes for these groups, after which the UniFrac distance matrix was represented through Principal Coordinate Analysis (PCoA). The R^2^ values obtained depending on the statistical test used were: Bray–Curtis R^2^ = 0.85, Unweighted UniFrac R^2^ = 0.73 and Weighted UniFrac R^2^ = 0.89 (these data are detailed in Table S6). These results support that microbiota diversity is significantly affected by the age of animals for both management systems (*p*-value = 0.001) (Figure 3). There is a notable difference between day-old chicks and the rest of the sampling moments for each group. However, mid-period and end sampling moments are also separated in PCoA graphics, indicating that microbiota diversity continued to increase, although to a lesser extent, until the end of the growing period.

## 4. Discussion

The present study assessed the caecal microbiota in two different management systems: fast-growing and slow-growing, with two different genetic broiler breeds, during their respective growing periods. In fact, knowing the main microbiota composition during the growing period and how management practices could influence its modulation could help quick decisions be taken at farm level [46,47]. In this sense, it might be interesting to consider microbiota composition as a biomarker of poultry health and productive performance [7,9,48]. It is well demonstrated that a greater complexity of the gut microbiota is observed as animals grow [49,50,51]. Our findings showed that there is an important change in microbiota composition from animals’ arrival to the mid-period, and a less pronounced variation has been observed from mid-period to the end of rearing. Microbiota becomes relatively stable at 21 days of rearing, coinciding with the gut maturation in broilers [26,27,49,50]. Although some authors reported that bacterial diversity in the intestinal tract is higher in breeds with high feed efficiency [9,52],the results of this study showed a similar microbiota diversity for both breeds through the production cycle [26,53]. This evidences the importance of flock management during the production cycle in terms of microbiota balance control [9,11,54]. It is important that any antibiotic alternative introduced in farms, such as feed additives or management practices, should promote microbiota development of phyla related to gut health and productive performance.

Regarding gut microbiota composition, the predominant phyla obtained in this study for both management systems were *Firmicutes* and *Bacteroidetes*, followed by *Proteobacteria* [9,27,50,55]. The colonization of the gastrointestinal tract starts at the moment of hatching [2,9,56]. During thefirst days, it becomes successively colonized by *Protebacteria*, specially by the *Enterobacteriaceae* family, and by *Firmicutes* [11]. Afterwards, *Firmicutes* dominate the caecal population, followed by *Bacteroidetes* [55,57]. *Firmicutes*, constitutes a heterogeneous phylum containing bacterial groups with different metabolic activities, and several studies have shown that a high level of this phylum is correlated with good intestinal health [58,59]. The *Bacteroidetes* phylum is stable through the growing period for both systems, playing an important role in converting fermentable starch to simple sugars and these, in turn, to volatile fatty acids to meet the energy demand of the host, so their presence could be particularly affected by diet components [49,56,60]. At the onset of rearing, *Proteobacteria* are also found in a high concentration for both groups. An increment of this phylum is associated with dysbiosis and, consequently, with an increase in the presence of zoonotic bacteria belonging to this phylum, such as *Salmonella* or *Campylobacter.* For this reason, it is important to ensure strict management practices at the start of the growing period, as any stress could produce an increase in this phylum, and could result in a higher shedding of pathogenic bacteria and environmental contamination throughout rearing [9,58,61,62]. It is an important concern for the poultry sector to maintain these bacteria under control from the beginning to the end of rearing, the last step before loading, transport and processing of chickens at the slaughterhouse. Nowadays, *Campylobacter* and *Salmonella* are still the two most important causes of zoonotic diseases in Europe, and poultry products are the main source of human infection [63].

At genus level, it is important to highlight that 75%, 93% and 97.8% are common to both management systems, at the beginning, mid- and end period, respectively. These results could suggest that, despite management practices in the field, the microbiota could have a similar development for both broiler production systems [26,64]. Moreover, although there exist some variations at genus level, the results obtained in terms of microbiota composition are broadly similar for both management systems. According to other authors, slight changes in microbiota composition have not always entailed a performance consequence [53,65].

The most predominant genera were *Oscillospira* spp., *Ruminococcus* spp., *Coprococcus* spp., *Lactobacillus* spp. and *Bacteroides* spp., in line with data reported by other authors [27,47,55]. These genera are associated with higher production rates, so it might be said that high levels of these genera are indicators of adequate intestinal health in poultry [5,6,47,50]. Among these, *Ruminococcus* spp. is known for its ability to degrade complex carbohydrates and thus may have contributed to an improved degradation of dietary fibre [66,67]. Moreover, *Lactobacillus* spp. is an important probiotic in promoting healthy gut, as these bacteria are believed to be responsible for starch decomposition and lactate fermentation [6,47,50,56]. In turn, *Bacteroidetes* spp. plays an important role in breaking down complex molecules to simpler compounds which are also essential for growth of the host and gut microbiota development [56,66]. In this aspect, feed has a vital influence on genus development [58,68,69,70]. In this study, fast-growing birds were fed a corn-based diet and slow-growing birds were fed a wheat-based one. Different studies support that diets based on barley or wheat instead of corn-based ones increase the prevalence of *Lactobacillus* spp. at caeca level [69,71,72], but these diets also could favour the proliferation of *Clostridium perfringens* and predispose young chicks to necrotic enteritis [68,73,74]. Nevertheless, corn- or soy-based diets could be deficient in available phosphorus and supplementation is often necessary [75]. Therefore, the most important aspect of diet management is to meet the metabolic requirements of animals by using a balanced diet formulation [36,68]. The application at field level of management techniques that produce the correct balance of any group of microorganisms that benefit intestinal health could result in animal health and productivity. Likewise, management techniques that favour the development of undesirable bacterial groups always need to be discarded.

In short, there are numerous factors that influence on microbiota composition development, and all of them should be valued globally in situ, according to their specific production characteristics [11]. Therefore, developing molecular techniques that can be applied in the field to measure the balance of the microbiota in each specific case could help us assess the impact of different management techniques on day-to-day work, and could be a promising line of research for our sector.

## 5. Conclusions

In conclusion, fast and slow-growing broiler microbiota is in constant development throughout rearing, being relatively stable at 21 days of age. *Firmicutes* and *Proteobacteria* are the most abundant phyla at the onset of the production cycle. However, while the *Firmicutes* increased their concentration for the two management systems throughout the growing period, the *Proteobacteria* decreased until the end of the cycle. Regarding the genus, it should be noted that the three most abundant groups for both systems, *R uminococcus* spp., *Lactobacillus* spp. and *Bacteroides* spp., are related to better productive performance and intestinal health.

## Figures and Tables

**Table 1:** Composition of starter and grower diets for fast-growing (FG) and slow-growing (SG) breeds.

Analytical Constituents (%)	Diet		
	Starter	Grower FG	Grower SG
Crude fat	3.5%	3.1%	3.8%
Crude protein	20.5%	19.4%	18.0%
Crude fibre	2.6%	3.1%	3.2%
Crude ash	6.6%	5.0%	5.5%
Lysine	1.14%	1.13%	0.94%
Methionine	0.62%	0.51%	0.40%
Calcium	1.00%	0.78%	1.00%
Phosphorus	0.69%	0.51%	0.43%
Sodium	0.15%	0.14%	0.17%
Ingredients	Corn, soy flour, wheat, soy oil, calcium carbonate, monocalcium phosphate, sodium chloride	Corn, soy flour, rice bran, calcium carbonate, sodium chloride	Wheat, soy flour, barley, soy oil, calcium carbonate, monocalcium phosphate, sodium chloride, sodium bicarbonate

**Table 2:** Weight of the animals (weight ± s.d) and conversion rate (CR ± s.d.) during the productive cycle for fast (FG) and slow-growing (SG) management systems.

	Fast-Growing (FG)		Slow-Growing (SG)	
Days of Life	Weight (g)	CR	Weight	CR
0	47.20 ± 0.98		41.31 ± 1.24	
7	184.80 ± 8.92	1.16 ± 0.10	146.05 ± 6.25	1.26 ± 0.14
14	492.90 ± 44.81	1.25 ± 0.18	368.23 ± 43.77	1.29 ± 0.35
21	823.32 ± 41.88	1.23 ± 0.16	547.21 ± 18.42	1.22 ± 0.63
28	1503.41 ± 77.66	1.30 ± 0.15	936.98 ± 31.20	1.34 ± 0.44
35	2043.72 ± 163.78	2.73 ± 0.74	1283.64 ± 93.16	2.70 ± 0.83
42	2605.91 ± 242.06	3.06 ± 1.30	1631.83 ± 105.98	3.29 ± 1.07
49			2049.22 ± 146.00	3.05 ± 1.08
56			2439.40 ± 183.25	3.17 ± 1.76
63			2776.33 ± 181.86	4.34 ± 1.99

**Table 3:** Alpha diversity according to management system (FG or SG) and sampling moment based on Chao 1 index.

SAMPLING TIME	FG	SG
Arrival day	88.3 ^a^	111.9 ^d^
Mid-period	384.4 ^b^	373.8 ^e^
End period	420.3 ^c^	447.2 ^f^

FG: Fast-growing breed. SG: Slow-growing breed. ^a,b,c^: Different superscripts within column FG indicate a significant difference within group (*p* ≤ 0.05). ^d,e,f^: Different superscripts within column SG indicate a significant difference within group (*p* ≤ 0.05).

**Table 4:** Taxonomic profiles at phylum level according to management system (FG or SG) and sampling moment based on MetagenomeSeq parametric test.

Breed	Fast-growing			Slow-growing		
Sampling Time	AD	MP	E	AD	MP	E
*Actinobacteria*	0.0%	0.3%	0.5%	0.2%	0.3%	0.4%
*Bacteroidetes*	5.0% ^a^	1.9% ^b^	5.7% ^c^	5.7% ^l^	1.9% ^m^	9.3% ^n^
*Cyanobacteria*	0.0% ^d^	0.5% ^d^	0.7% ^e^	0.0%	0.4%	1.1%
*Firmicutes*	58.6% ^f^	95.1% ^g^	90.3% ^h^	61.1%^o^	95.2% ^p^	85.6% ^q^
*Proteobacteria*	36.4% ^i^	1.3% ^j^	1.5% ^k^	32.8% ^r^	1.2% ^s^	1.7% ^s^
*Tenericutes*	0.0%	0.3%	0.6%	0.2%	0.4%	1.1%
Unassigned; NA	0.0%	0.6%	0.8%	0.0%	0.6%	0.8%

AD: Arrival day, MP: Mid-period, E: End. ^a–k^: Different superscripts indicate a significant difference within each phylum during rearing for fast-growing management system (*p* ≤ 0.05). ^l–s^: Different superscripts indicate a significant difference within each phylum during rearing for slow-growing management system (*p* ≤ 0.05).

**Table 5:** Taxonomic profiles at genus level according to sampling moment in fast-growing management system.

Phylum	Family	Genus	AD	MP	E
Unassigned			0.0%	0.6%	0.8%
*Bacteroidetes*	*Bacteroidaceae*	*Bacteroides*	1.5%	0.5%	3.1%
	*Porphyromonadaceae*	*Parabacteroides*	1.2%	0.4%	0.7%
	*Rikenellaceae*	-	2.0%	1.1%	1.2%
	*Odoribacteraceae*	*Butyricimonas*	0.3%	0.0%	0.7%
*Cyanobacteria*	-	-	0.0%	0.5%	0.7%
*Firmicutes*	*Planococcaceae*	-	0.0%	0.5%	0.4%
	*Enterococcaceae*	-	3.7%	0.0%	0.0%
		*Enterococcus*	3.0%	0.2%	0.1%
	*Lactobacillaceae*	*Lactobacillus*	0.9%	3.9%	2.8%
	-	-	0.2%	0.5%	0.6%
	-	-	13.7%	29.4%	28.9%
	*Christensenellaceae*	-	0.0%	0.2%	0.6%
	*Clostridiaceae*	-	0.6%	0.0%	0.3%
		-	5.6%	0.2%	0.2%
		*Clostridium*	4.1%	0.5%	0.5%
	*Lachnospiraceae*	-	4.9%	10.4%	10.2%
		*Blauria*	0.7%	2.0%	2.1%
		*Coprococcus*	1.6%	4.0%	3.5%
		*Dorea*	0.2%	1.4%	1.1%
		*Epulopscium*	2.6%	0.0%	0.0%
		*[Ruminococcus]*	2.5%	3.3%	2.9%
	*Ruminococcaceae*	-	5.7%	18.1%	17.7%
		*Anaerotruncus*	0.0%	0.5%	0.4%
		*Faecalibacterium*	0.9%	1.5%	2.0%
		*Oscillospira*	3.5%	9.6%	8.8%
		*Ruminococcus*	2.1%	5.0%	4.4%
	*Erysipelotrichaceae*	-	0.9%	0.9%	0.4%
		*Coprobacillus*	0.4%	0.9%	0.5%
		cc_115	0.0%	0.9%	0.6%
*Proteobacteria*	*Enterobacteriaceae*	-	36.4%	1.3%	1.5%

AD: arrival day, MP: mid-period, E: end.

**Table 6:** Taxonomic profiles at genus level according to the sampling moment in slow-growing management system.

Phylum	Family	Genus	AD	MP	E
Unassigned			0.0%	0.6%	0.8%
*Bacteroidetes*	*Bacteroidaceae*	*Bacteroides*	2.6%	0.4%	4.1%
	*Porphyromonadaceae*	*Parabacteroides*	1.0%	0.5%	1.1%
	*Rikenellaceae*	-	2.0%	1.1%	3.1%
	*Odoribacteraceae*	*Butyricimonas*	0.0%	0.0%	1.1%
*Cyanobacteria*			0.0%	0.4%	1.1%
*Firmicutes*	*Planococcaceae*	-	0.2%	0.5%	0.4%
	*Enterococcaceae*	-	3.6%	0.0%	0.0%
		*Enterococcus*	1.0%	0.2%	0.4%
	*Lactobacillaceae*	*Lactobacillus*	1.2%	3.4%	2.9%
	-	-	0.4%	0.6%	0.3%
	-	-	14.6%	29.9%	30.0%
	*Clostridiaceae*	-	4.8%	0.2%	0.3%
		*Clostridium*	2.7%	0.4%	0.4%
	*Lachnospiraceae*	-	6.5%	10.3%	8.6%
		*Blauria*	0.8%	1.8%	1.5%
		*Coprococcus*	1.6%	3.8%	3.2%
		*Dorea*	0.8%	1.3%	0.7%
		*Epulopscium*	2.4%	0.0%	0.0%
		*Ruminococcus*	2.1%	3.3%	2.3%
	*Ruminococcaceae*	-	7.5%	18.4%	17.0%
		*Anaerotruncus*	0.0%	0.5%	0.3%
		*Faecalibacterium*	1.5%	1.8%	1.5%
		*Oscillospira*	5.8%	9.6%	7.7%
		*Ruminococcus*	1.7%	5.1%	3.6%
	*Erysipelotrichaceae*	-	1.0%	0.9%	0.6%
		*Coprobacillus*	0.4%	0.9%	0.5%
		cc_115	0.0%	0.8%	0.6%
*Proteobacteria*	*Enterobacteriaceae*	-	32.6%	1.2%	0.9%
*Tenericutes*	-	-	0.2%	0.4%	0.7%

AD: Arrival day; MP: Mid-period; E: End.

## Supplementary Materials

The following are available online at https://www.mdpi.com/2076-2615/10/8/1401/s1, Table S1. Statistical comparison of alpha diversity between sample groups based on Chao 1 index, Table S2. Statistical comparation of alpha diversity between sample groups based on Shannon index, Table S3. Statistical comparation of alpha diversity between sample groups based on Simpson index, Table S4. Statistical comparation of alpha diversity between sample groups based on Observed OTUs index, Table S5. Different taxonomic profiles at genus level according to the moment of the growing period in fast (FG) and slow-growing (SG) breeds, Table S6. Statistical comparison between beta diversity indexes calculated according to the different methods.
