# Supplementary material for: Fast and Slow-Growing Management Systems: Characterisation of Broiler Caecal Microbiota Development throughout the Growing Period
Source: Animals (Basel). 2020 Aug 12;10(8):1401. doi: 10.3390/ani10081401 (PMC7459849; doi:10.3390/ani10081401)
Supplement: Supplementary file 1 [file animals-10-01401-s001.pdf]

Table S1. Statistical comparation of alpha-diversity between sample groups based on Chao 1 index.

Group1	Group2	Group1 mean	Group1 std	Group2 mean	Group2 std	t stat	p-value
FGAD	FGMP	88.32	29.03	384.44	16.50	-23.41	1.11e-11
FGMP	FGE	384.42	16.50	420.31	17.86	-4.43	0.00
SGAD	SGMP	111.86	8.60	373.82	15.96	31.93	2.93e-13
SGMP	SGE	373.86	15.96	447.22	4.66	-13.24	1.69e-10

FGAD: fast-growing breed at arrival day; FGMP: fast-growing breed at mid-period; FGE: fast-growing breed at the end of the growing period; SGAD: slow-growing breed at arrival day; SGMP: slow-growing breed at mid-period; SGE: slow-growing breed at the end of the growing period.

Table S2. Statistical comparation of alpha-diversity between sample groups based on Shannon index.

Group1	Group2	Group1 mean	Group1 std	Group2 mean	Group2 std	t stat	p-value
FGAD	FGMP	1.29e11	0.19	6.69e11	0.21	-4.55e11	2.53e04
FGMP	FGE	6.69e11	0.21	6.21e11	0.23	4.70e11	0.00
SGAD	SGMP	1.51e11	0.13	6.69e11	0.15	6.25e11	0.00
SGMP	SGE	6.69e11	0.15	6.52e11	0.18	2.16e11	0.06

FGAD: fast-growing breed at arrival day; FGMP: fast-growing breed at mid-period; FGE: fast-growing breed at the end of the growing period; SGAD: slow-growing breed at arrival day; SGMP: slow-growing breed at mid-period; SGE: slow-growing breed at the end of the growing period.

Table S3. Statistical comparation of alpha-diversity between sample groups based on Simpson index.

Group1	Group2	Group1 mean	Group1 std	Group2 mean	Group2 std	t stat	p-value
FGAD	FGMP	0.36	0.06	0.98	0.01	-3.03e11	4.06e02
FGMP	FGE	0.98	0.01	0.96	0.01	3.87e11	0.00
SGAD	SGMP	0.53	0.02	0.98	0.00	5.48e11	1.67e04
SGMP	SGE	0.98	0.00	0.97	0.00	4.04e11	0.00

FGAD: fast-growing breed at arrival day; FGMP: fast-growing breed at mid-period; FGE: fast-growing breed at the end of the growing period; SGAD: slow-growing breed at arrival day; SGMP: slow-growing breed at mid-period; SGE: slow-growing breed at the end of the growing period.

Table S4. Statistical comparation of alpha-diversity between sample groups based on Observed OTUs index.

Group1	Group2	Group1 mean	Group1 std	Group2 mean	Group2 std	t stat	p-value
FGAD	FGMP	41.18	1.05e10	374.46	1.72e10	-3.70e11	3.09e03
FGMP	FGE	374.46	1.72e10	407.56	1.93e11	-3.84e11	0.00
SGAD	SGMP	46.92	5.28e11	363.38	1.60e11	4.01e11	2.55e03
SGMP	SGE	363.38	1.60e11	437.72	5.55e10	-1.31e11	1.85e00

FGAD: fast-growing breed at arrival day; FGMP: fast-growing breed at mid-period; FGE: fast-growing breed at the end of the growing period; SGAD: slow-growing breed at arrival day; SGMP: slow-growing breed at mid-period; SGE: slow-growing breed at the end of the growing period.

Table S5. Different taxonomic profiles at genus level according to the moment of the growing period in fast (FG) and slow-growing (SG) breeds.

Sampling moment	Breed	Phylum	Class	Order	Family	Genus	Percentage
**Arrival day**	**FG**	*Bacteroidetes*	*Bacteroidia*	*Bacteroidales*	*[Odoribacteraceae]*	*Butyricimonas*	0.27%
		Firmicutes	*Clostridia*	*Clostridiales*	*Peptostreptococcaceae*		0.22%
		Firmicutes	*Clostridia*	*Clostridiales*	*Lachnospiraceae*	*Lachnospira*	0.45%
	**SG**	*Actinobacteria*	*Actinobacteria*	*Bifidobacteriales*	*Bifidobacteriaceae*	*Bifidobacterium*	0.20%
		Firmicutes	*Bacilli*	*Bacillales*	*Planococcaceae*	NA	0.20%
		Firmicutes	*Clostridia*	*SHA-98*	-	-	0.24%
		Proteobacteria	*Betaproteobacteria*	*Burkholderiales*	*Alcaligenaceae*	*Sutterella*	0.20%
		Tenericutes	*Mollicutes*	*RF39*	-	-	0.19%
**Mid-period**	**FG**	*Firmicutes*	*Clostridia*	*SHA-98*	-	-	0.02%
		Proteobacteria	*Betaproteobacteria*	*Burkholderiales*	*Alcaligenaceae*	*Sutterella*	0.01%
	**SG**	*Proteobacteria*	*Alphaproteobacteria*	*RF32*	-	-	0.01%
**End**	**SG**	*Firmicutes*	*Clostridia*	*Clostridiales*	*Lachnospiraceae*	*Epulopiscium*	0.01%

Table S6. Statistical comparation between beta-diversity indexes calculated according the different methods.

Beta-diversity matrix	Adonis test			ANOSIM	
	F-stat	R^2	p-value	Statistic value	p-value
Bray-Curtis	49.076	0.84795	0.001	0.79932682926829257	0.001
Unweighted-Unifrac	23.453	0.72716	0.001	0.70287804878048776	0.001
Weighted-Unifrac	69.523	0.88764	0.001	0.82837073170731712	0.001
